# Endoscopic esophageal reconstruction in epiphrenic diverticulum: septotomy-free diverticulectomy and suturing

**DOI:** 10.1016/j.vgie.2025.10.010

**Published:** 2025-10-30

**Authors:** Fatih Aslan, Serhat Ozer, Afak Durur Karakaya, Selman Sogut

**Affiliations:** 1 Koc University Hospital, Gastroenterology and Advanced Endoscopy, Istanbul, Turkiye; 2 Koc University Hospital, Radiology, Istanbul, Turkiye; 3 Koc University Hospital, Anesthesiology and Reanimation, Istanbul, Turkiye

## Abstract

**Background and Aims:**

Epiphrenic diverticulum (ED) is a rare pulsion-type sac that typically develops secondary to esophageal motility disorders. Although surgical resection has been the standard treatment, advances in third-space endoscopy have introduced minimally invasive alternatives. We describe an endoscopic approach combining peroral endoscopic myotomy (POEM) with septotomy-free diverticulectomy and endoscopic suturing.

**Methods:**

A 65-year-old man with spastic achalasia and ED underwent selective circular myotomy using the POEM technique. The diverticulum was inverted into the lumen, its neck closed with barbed sutures at the muscular layer, and subsequently resected from the luminal side using a polypectomy snare. The mucosal defect was closed with additional suturing.

**Results:**

The procedure was completed without adverse events. Postoperatively, the diverticulum completely disappeared, and the patient's symptoms resolved. Follow-up endoscopy confirmed complete healing with no recurrence.

**Conclusions:**

This combined technique restores the tubular anatomy of the esophagus and maintains luminal patency. Septotomy-free diverticulectomy with endoscopic suturing may represent a minimally invasive and effective alternative to surgery for select patients with EDs.

## Introduction

Epiphrenic diverticula (EDs), frequently accompanied by a motility disorder, are rare pulsion-type sacs seen in the distal esophagus. They are mostly located within the distal 10 cm of the esophagus, approximately 4 to 8 cm proximal to the gastroesophageal junction (GEJ). They occur as a result of herniation of the mucosa, muscularis mucosa, and submucosa layers toward the mediastinum through an area of weakness in the muscularis propria layer.^1^

Although the traditional treatment approach has been surgical resection, significant advancements have been made in third-space endoscopy over the last decade. In this context, peroral endoscopic myotomy (POEM) offers an alternative therapeutic option.^2^^,^^3^

In this article, we present a novel minimally invasive endoscopic approach and treatment technique applied to a symptomatic ED case, with the pathophysiological basis explained.

## Case

**Figure 1:**
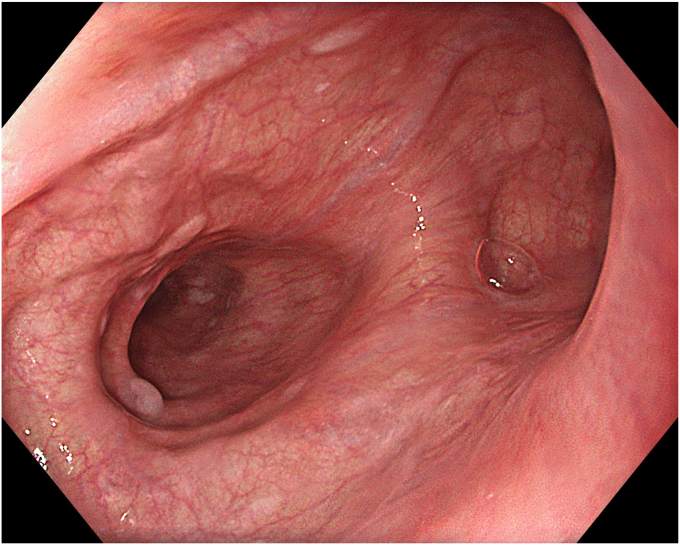
Endoscopic view of the epiphrenic diverticulum at the right lateral wall of the distal esophagus.

A 65-year-old male patient presented with dysphagia, chest pain, cough, and vomiting. Esophagography and thoracic computed tomography revealed an ED of approximately 4 cm in diameter, with regular margins and a filling defect, located on the right lateral wall of the distal esophagus. In proximal and distal sides adjacent to the diverticulum, radiologic features were consistent with spastic achalasia. Endoscopy also demonstrated a diverticulum of approximately 4 cm in diameter proximal to the tight-looking GEJ (Fig. 1). High-resolution esophageal manometry was performed and consistent with spastic achalasia. Before the procedure, the patient's dysphagia score was 3 and the Eckardt score was 8.

A POEM combined with endoscopic diverticulectomy was planned after informed consent was obtained. Prophylactic intravenous piperacillin-tazobactam (4 × 4.5 g/d) and a proton pump inhibitor were administered and continued until discharge.

With the patient under general anesthesia, a standard gastroscope was inserted. Approximately 8 cm proximal to the diverticulum and right above the spastic segment, a submucosal injection of 0.9% NaCl mixed with a small amount of indigo carmine was performed using a sclerotherapy needle (NeedleMaster, Olympus, Tokyo, Japan) at the 5-o'clock position. An endoscopic hood (Olympus) and a knife (Triangle Knife Jet, Olympus) were used. A linear mucosal incision was made to reach the submucosal space with settings of PulseCut Slow (40 W, effect 2) and SprayCoag (40 W, effect 2).

**Figure 2:**
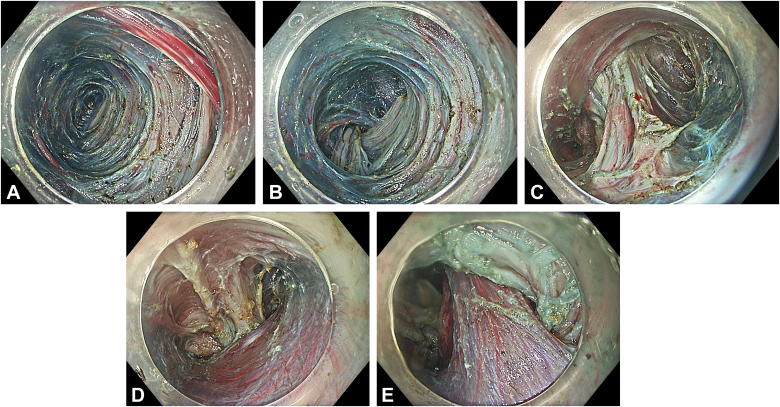
**A,** Endoscopic view of the submucosal tunnel in the esophageal body. **B,** Endoscopic view of the submucosal tunnel distal to the gastroesophageal junction. **C,** Endoscopic view of the posterior margin of the diverticular opening from the posterior tunnel. **D,** Endoscopic view of the anterior margin of the diverticular opening from the anterior tunnel. **E,** Endoscopic view of both anterior and posterior margins of the diverticular opening; the anterior and posterior submucosal tunnels merged within the diverticulum.

**Figure 3:**
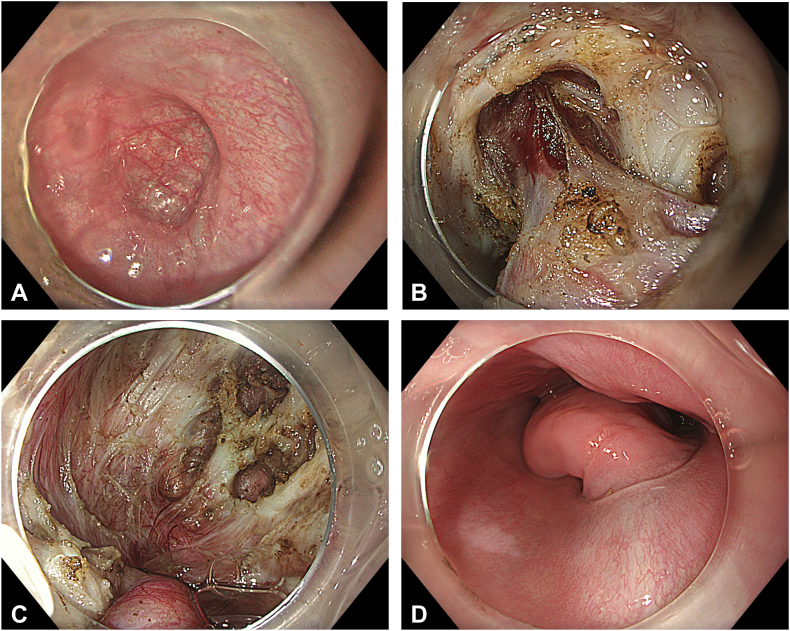
**A,** Endoscopic luminal view of the diverticular base before complete inversion. **B,** Endoscopic submucosal view of the diverticular base before complete inversion. **C,** Endoscopic submucosal view of the diverticular base after complete inversion. **D,** Completely inverted epiphrenic diverticulum, luminal view.

**Figure 4:**
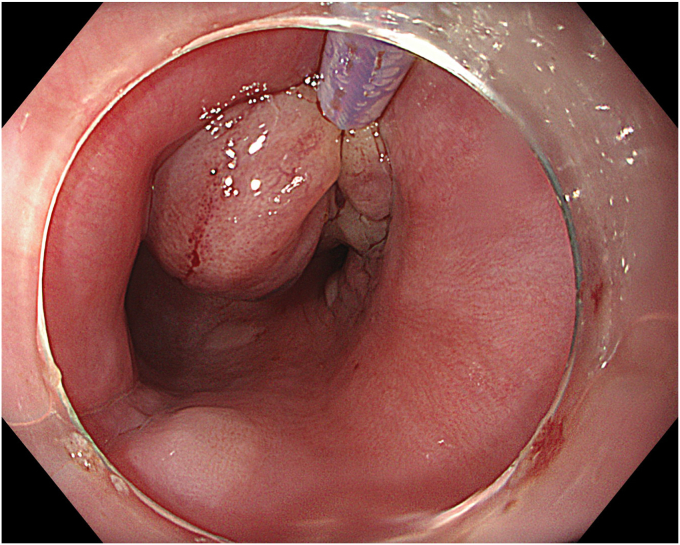
The inverted diverticulum being retracted with a snare.

**Figure 5:**
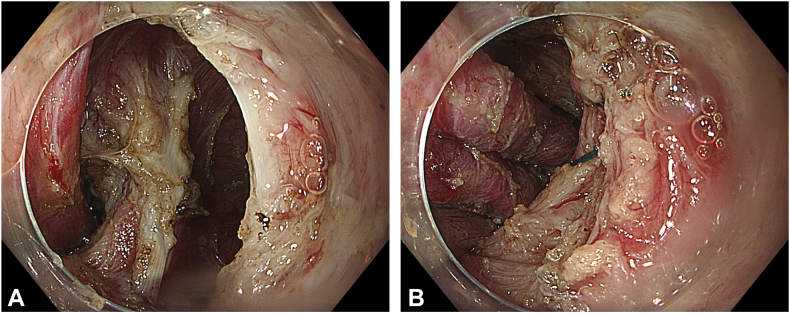
**A,** Endoscopic submucosal view of the diverticular opening. **B,** Endoscopic submucosal view of the diverticular opening after closure with barbed suture.

**Figure 6:**
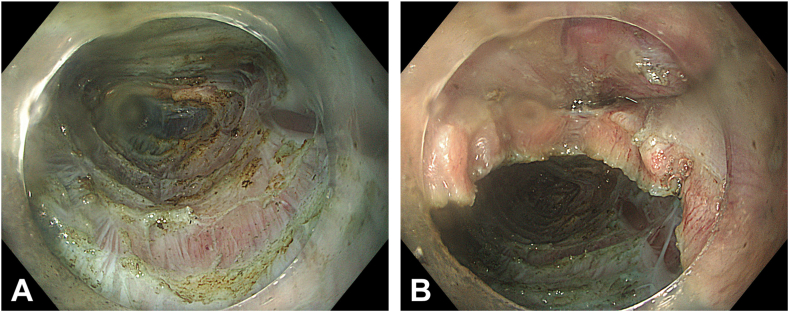
**A,** Endoscopic view of the intermuscular tunnel, showing the circular and longitudinal muscularis propria at the upper and lower sides, respectively. **B,** Selective circular myotomy.

**Figure 7:**
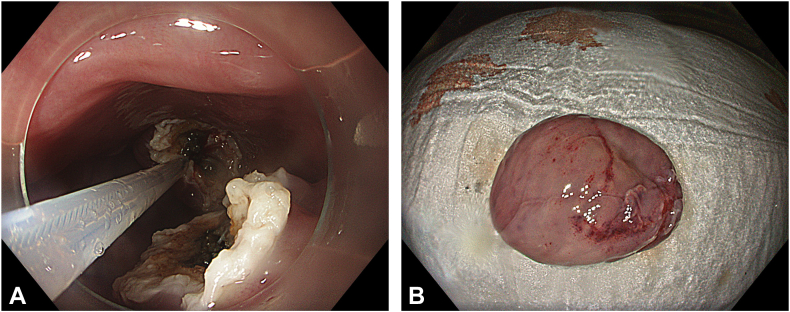
**A,** Endoscopic diverticulectomy of the inverted diverticulum. **B,** Macroscopic view of the diverticulectomy specimen.

**Figure 8:**
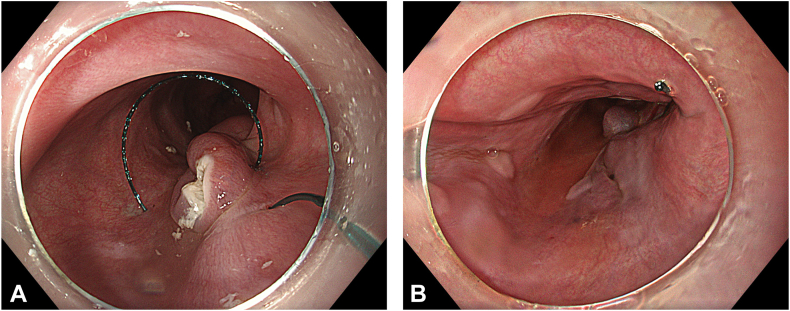
**A,** Endoscopic view of the closed mucosal defect. **B,** Endoscopic view of the reconstructed esophagus.

**Figure 9:**
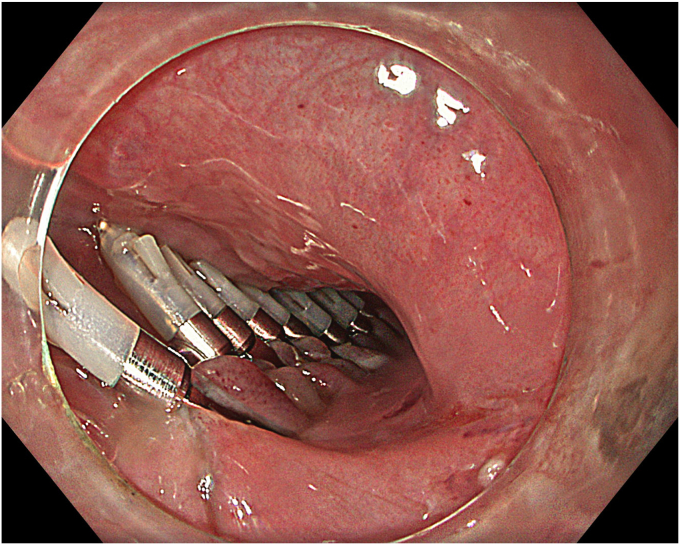
Endoscopic view of closed tunnel entry.

Two separate submucosal tunnels, from the posterior and anterior sides proximal to the ED, were created. The tunnels were merged in both the proximal and distal sides of the diverticulum (Fig. 2). Some spots of the diverticulum could not be reached with a standard endoscope, and a nasal endoscope with a needle-knife (Cook Medical, Winston-Salem, NC, USA) was used to complete the dissection. The diverticulum was fully freed, mobilized, and inverted into the lumen (Fig. 3). Then, a 30-mm standard polypectomy snare (Steris, Dublin, Ireland) was advanced in outside the endoscope and opened to cover the diverticulum from the luminal side. Grasping forceps (Ovesco, Tübingen, Germany) were used to pull the diverticulum inside the open snare, and the snare was closed (Fig. 4). The endoscope was removed as the snare was kept closed with gentle manual traction. This maneuver prevented the inverted diverticulum from returning to its previous and original position and maintained tunnel patency, facilitating subsequent steps. Next, barbed sutures (V-Loc, Medtronic Ltd, Dublin, Ireland) were delivered into the tunnel using a needle holder (SutuArt, Olympus) through the working channel, and suturing, selectively on the muscular layer at the diverticular neck, was completed. Excess suture material was cut and removed using endoscopic scissors (Loop Cutter, Olympus) (Fig. 5). Subsequently, a selective circular posterior myotomy was performed between the proximal margin of the diverticulum and just above the GEJ, without interfering with the captured diverticulum, followed by a full-thickness myotomy across the GEJ. To enhance visualization and ensure safe separation of the muscle layers, we injected a small amount of saline mixed with indigo carmine into the intermuscular plane between the circular and longitudinal fibers, which facilitated a more controlled and selective myotomy (Fig. 6). In the final stage, the already inverted diverticulum was resected (Fig. 7), and the subsequent mucosal defect was closed with barbed sutures (Fig. 8). The final touch, closure of the tunnel entry, was completed with hemostatic clips (Olympus) (Fig. 9).

No intraoperative adverse event occurred. Intra-abdominal CO_2_ accumulation, an anticipated consequence rather than an adverse event, reflected by increased peak airway pressure and significant abdominal distension, was managed by percutaneous decompression with a 14-gauge needle inserted 2 fingers above the umbilicus.

Oral intake was held for the first 2 postoperative days, and intravenous fluids with total parenteral nutrition were administered. A follow-up chest radiograph revealed minimal atelectasis and pleural effusion, which resolved with medical management. On day 3, the patient began a liquid diet, and he was on a regular diet by day 5. Follow-up esophagography demonstrated complete resolution of the diverticulum with no contrast retention. The patient was discharged uneventfully on day 5.

**Figure 10:**
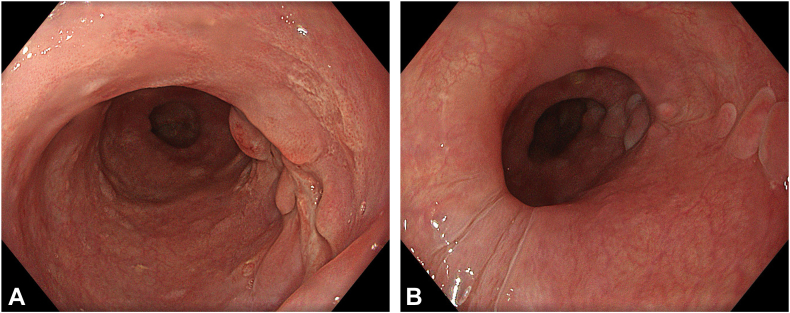
**A,** Scar tissue in diverticulectomy site by third month. **B,** Endoscopic view of the tunnel entry and reconstructed esophagus by third month.

Histopathologic analysis confirmed as expected that the resected specimen included mucosa, muscularis mucosa, and submucosa, in a way consistent with a diverticulum. At the 3-month follow-up endoscopy, the sutures had fully dissolved with scar tissue at the diverticulectomy site (Fig. 10). Both the dysphagia score and the Eckardt score were 0. There was neither evidence of recurrence nor food retention, and the patient was doing well, with all symptoms completely resolved (Video 1, available online at www.videogie.org).

## Discussion

EDs are pulsion-type sacs that develop as a result of increased intraluminal pressure, mostly secondary to motility disorders, and typically occur in weak anatomical spots at the right or left lateral wall of the distal esophagus.^1^ Traditional surgical treatment methods, particularly thoracotomy, have been associated with high morbidity and recurrence rates.^2^^,^^4^ In recent years, however, less-invasive alternative therapeutic approaches, such as POEM, have emerged with the advancement of third-space endoscopy techniques. Nonetheless, despite technical success, clinical outcomes reported in the literature remain variable.^3^^,^^5^, ^6^, ^7^

In contrast to normal swallowing physiology,^8^ when the luminal diameter increases or simultaneous peristalsis is impaired, intraluminal pressure cannot be evenly distributed. In such situations, food progresses from the esophagus to the stomach only with the aid of gravity, liquid intake, and postural adjustments.^9^^,^^10^ In spastic esophageal motility disorders (eg, type III achalasia, nutcracker esophagus, hypercontractile motility disorders), intraluminal pressure distribution is irregular, and in the absence of simultaneous peristaltic waves, abnormal intraluminal pressure elevations occur within the esophageal lumen. Therefore, both motility-related abnormal intraluminal pressure and food stasis in the esophageal lumen generate additional wall stress, predisposing to diverticulum formation in anatomically weak areas.^11^^,^^12^

**Figure 11:**
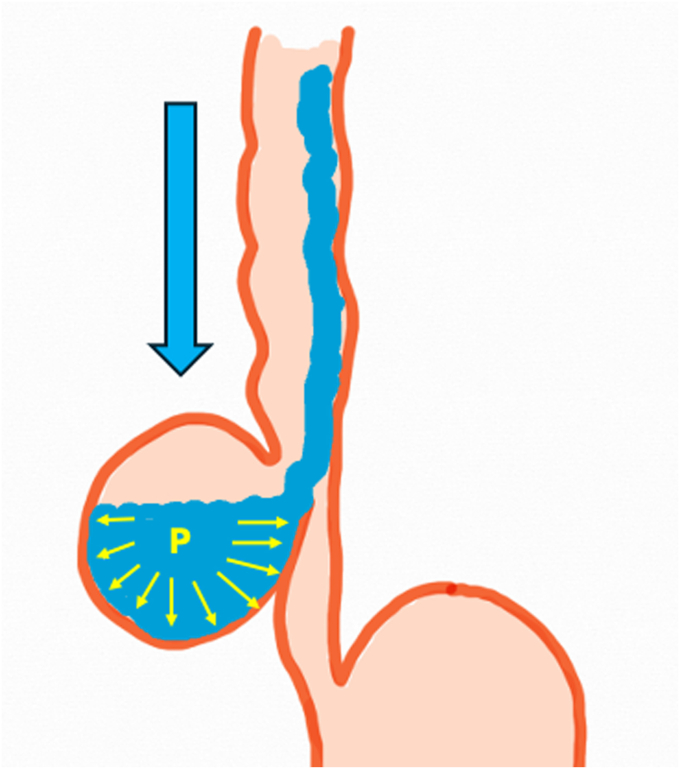
Luminal narrowing secondary to increased intradiverticular pressure.

Initially formed diverticula may partially reduce intraluminal pressure; however, over time, they function like reservoirs that retain food for prolonged periods, which leads to increased intradiverticular pressure. This progressive rise in pressure facilitates diverticular enlargement and creates a vicious cycle that is difficult to reverse.^13^ Moreover, as the diverticulum enlarges, ingested solids and liquids tend to enter it. Advanced food retention further increases diverticular volume and exerts additional pressure on the esophageal lumen. Thus, the diverticulum begins to act like a valve by narrowing or closing the esophageal lumen, thereby limiting the emptying of both the esophagus and the diverticular content. This condition may result in advanced-stage symptoms such as vomiting and weight loss (Fig. 11).^4^^,^^14^^,^^15^

In patients with ED accompanied by motility disorders, POEM with or without septotomy has been reported to achieve favorable technical and clinical outcomes.^3^^,^^5^^,^^6^^,^^16^ However, when the diverticulum remains intact, it continues to function as a reservoir.^17^^,^^18^ Even if septotomy is performed to facilitate diverticular emptying, the absence of peristalsis causes drainage to occur solely by gravity. Consequently, if no corrective intervention is applied to the diverticulum and it continues to persist as a reservoir, food retention within the diverticulum remains inevitable despite positional changes, and symptoms may continue intermittently, albeit reduced. Therefore, long-term outcomes of POEM with or without septotomy with EDs are required, and this remains crucial to resolving this clinical dilemma.

**Figure 12:**
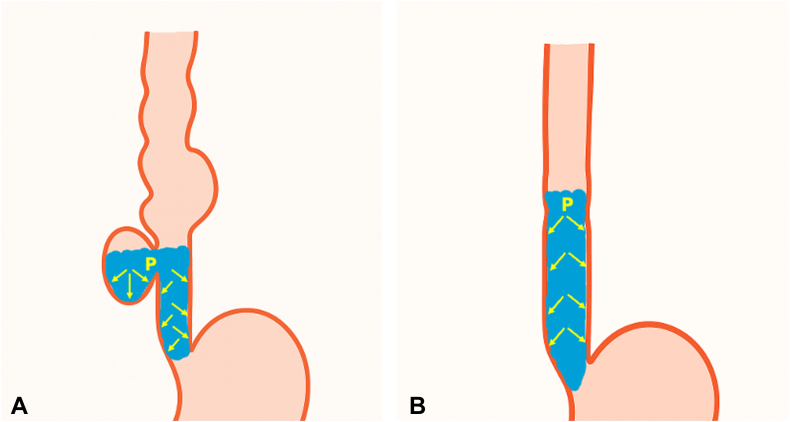
**A,** Pretreatment: accumulation of liquids and solid food increases intradiverticular and intraesophageal intraluminal pressure. **B,** Post-treatment: restoration of the tubular anatomy balances intraluminal pressure and increases flow velocity with gravitational force.

The pathophysiology of ED can be explained within the framework of classical pathophysiological principles.^19^^,^^20^ Disturbances in intraluminal pressure distribution, particularly in the presence of esophageal dilatation and motility disorders, may increase wall stress and contribute to the development and progression of diverticula. Therefore, preserving the tubular anatomy of the esophagus and maintaining balanced intraluminal pressure transmission may support long-term clinical success (Fig. 12).

In our case, the diverticulum was mobilized, inverted into the lumen, and subsequently resected. The resulting mucosal defect was closed with absorbable barbed sutures. This reconstruction aimed to ensure balanced transmission of pressure along the tubular system, thereby facilitating smooth passage of ingested food into the stomach. In addition, by eliminating the reservoir that diverted intraluminal contents away from the natural lumen, we established a reconstructed pathway that promotes forward flow within the esophagus. Resection of the inverted diverticulum was also considered important to prevent potential scenarios such as pressure imbalance or intermittent dysphagia.

Myotomy was initiated at the proximal margin of the diverticulum (spastic segment) with the goal of reducing abnormal intraluminal pressure. Furthermore, with the decrease of the pressure gradient to be overcome in the distal segment, the procedure was expected to enhance clinical efficacy and support long-term success on a physiopathological basis.

EDs are typically located in the distal esophagus, most commonly at the 9- or 3-o'clock position.^1^ Because external wall support is limited in these regions, myotomy was extended from the spastic segment proximal to the diverticulum to just beyond the GEJ to prevent new diverticulum formation and to balance intraluminal pressure distribution. To provide additional anatomic support, we performed the myotomy posteriorly at the 5 to 6 o’clock position, corresponding to the vertebral axis and the descending aorta.^21^

Unlike the classical approaches described in the literature, an intermuscular tunnel was created between the circular and longitudinal muscle layers, which facilitated selective cutting of the circular muscle. Based on our experience, performing the myotomy at the posterior 5- to 6-o'clock position may represent a safe and feasible option owing to the anatomic support provided by the vertebral column and descending aorta. This approach may contribute to preserving the tubular anatomy of the esophagus and preventing the formation of new diverticula.

Another notable feature of this case is that reconstruction was achieved without septotomy, solely by closing the diverticular neck. Unlike our previous cases,^7^ no septomyotomy was performed on the diverticular septum; instead, sutures were placed in alignment with the circular muscle layer. The diverticular neck was closed with sutures applied from distal to proximal. To preserve luminal patency and prevent potential clinical symptoms such as intermittent dysphagia, we resected the inverted diverticulum using a standard polypectomy technique. This approach was considered to ensure continuity of the lumen and to help balance intraluminal pressures. Following diverticulectomy, the resulting mucosal defect was closed with absorbable barbed sutures; suturing of both the diverticular neck and the mucosal defect was thought to provide a robust reconstruction.

In our opinion, although objective measurements demonstrating pressure changes were not available, based on physiological principles, myotomy, diverticulectomy, and closure may preserve esophageal integrity and balance intraluminal pressure distribution.^20^ This approach may increase intraluminal flow (solid food, saliva, and liquids), reduce wall stress, and thereby improve esophageal clearance while helping prevent the formation of new diverticula (Fig. 12). Follow-up endoscopy and clinical evaluation in our case confirmed preservation of the tubular anatomy of the esophagus and complete resolution of symptoms.

In third-space endoscopic procedures such as endoscopic submucosal dissection, POEM, endoscopic full-thickness resection, and submucosal tunneling endoscopic resection, CO_2_ diffuses rapidly through tissue planes, leading to intra-abdominal free air.^22^, ^23^, ^24^ This can occasionally progress to tension pneumoperitoneum or subcutaneous emphysema if not promptly managed. However, these events are predictable, usually mild, and easily controlled with close anesthesia monitoring and timely needle decompression.^25^ In our case, although intra-abdominal distension developed during the procedure, it was anticipated and promptly relieved by percutaneous decompression using a 14-gauge needle inserted 2 fingers above the umbilicus. The procedure was then safely completed without hemodynamic instability or other adverse events.

In our case, the applied technique has both positive and limiting aspects. As in our previous experience,^7^ it is considered to contribute to the reconstruction of the tubular structure of the esophagus. In addition, being a minimally invasive endoscopic method, it allows for a faster recovery process. Considering the approximately 1-year follow-up period, a stable and symptom-free course has been observed both clinically and endoscopically. However, some limitations also exist. Although we did not encounter them in this case, as with other advanced endoscopic procedures, risks such as bleeding, dehiscence of the suture line, and leakage are possible. Furthermore, the fact that this method has so far been applied in only a single case and has not yet been tested in larger patient series is considered an important limitation.

In conclusion, the combination of septotomy-free POEM with endoscopic diverticulectomy and suturing may be considered a safe and effective therapeutic option with EDs. With the integration and increasing applicability of endoscopic suturing techniques, it can be anticipated that complex cases such as EDs may potentially be managed endoscopically. These techniques may be regarded as a potential alternative for expanding therapeutic options in patients who were previously thought to require surgical approaches.

## Patient consent

Written informed consent was obtained from the patient for the procedure.

## Disclosure

All authors disclosed no financial relationships.

## Supplementary data

Video 1 Endoscopic esophageal reconstruction for epiphrenic diverticulum: a septotomy-free diverticulectomy and suturing (7.58 minutes).
